# Supplementary material for: Causes and trends in liver disease and hepatocellular carcinoma among men and women who received liver transplants in the U.S., 2010-2019
Source: PLoS One. 2020 Sep 18;15(9):e0239393. doi: 10.1371/journal.pone.0239393 (PMC7500679; doi:10.1371/journal.pone.0239393)
Supplement: S1 Table — (DOCX) [file pone.0239393.s001.docx]

**S1 Table. Diagnosis categorization criteria (methodology).**

| **Condition** | **Diagnosis Inclusion Criteria** | **Serology Inclusion Criteria** |
| --- | --- | --- |
| HCV | Cirrhosis: Type C, Alcoholic cirrhosis with hepatitis C, Acute hepatitis necrosis: Type C, Hepatitis C: Chronic or Acute | HCV sero-positive |
| ALD | Acute alcoholic hepatitis, alcoholic cirrhosis without HCV |  |
| NAFLD | Cirrhosis: fatty liver (NASH, nonalcoholic steatohepatitis) |  |
| Cholestatic | Primary biliary cirrhosis, secondary biliary cirrhosis (Caroli's disease, choledochal cyst, or other), primary sclerosing cholangitis (Crohn's disease, ulcerative colitis, no bowel disease, other), familial cholestasis (Byler's disease, other), Cholestatic liver disease, biliary atresia, biliary hypoplasia, neonatal cholestatic liver disease |  |
| HBV | Cirrhosis: Type B, Cirrhosis: type B and D, Acute hepatitis necrosis: Type B, Acute hepatitis necrosis: Type B and D, Hepatitis B: Chronic or Acute | HBsAg-positive |
| Cryptogenic | Cryptogenic cirrhosis |  |
| Autoimmune | Cirrhosis: autoimmune |  |
| Metabolic | Cystic fibrosis, A1AD, Wilson’s disease, hemochromatosis, glycogen storage disease, hyperlipidemia, tyrosinemia, oxaluria, maple syrup urine disease, other metabolic disease |  |
| Acute hepatic necrosis (non-HBV/HCV) | AHN: drug, AHN: Type A, AHN: other, AHN: etiology unknown |  |
| Unspecified causes of HCC | Fibrolamellar carcinoma, hepatocellular carcinoma (HCC) with cirrhosis, HCC without cirrhosis |  |
| Graft failure | Graft-vs-host disease, graft failure |  |
| Benign hepatic tumors | Hepatic adenoma, polycystic liver disease, other benign tumor |  |
| Other malignant liver tumors | Cholangiocarcinoma, hepatoblastoma, hemangioendothelioma, hemangiosarcoma, angiosarcoma, bile duct cancer, secondary hepatic malignancies |  |
| Budd-Chiari | Budd-Chiari Syndrome |  |
| Miscellaneous | Congenital hepatic fibrosis, total parenteral nutrition or hyperalimentation induced liver disease, trauma, drug/industry related cirrhosis, cirrhosis type A |  |
| Unspecified causes of liver disease/cirrhosis | chronic active hepatitis of unknown etiology, other cause of cirrhosis, unknown causes |  |
