# Supplementary material for: Causes and trends in liver disease and hepatocellular carcinoma among men and women who received liver transplants in the U.S., 2010-2019
Source: PLoS One. 2020 Sep 18;15(9):e0239393. doi: 10.1371/journal.pone.0239393 (PMC7500679; doi:10.1371/journal.pone.0239393)
Supplement: S2 Table — (DOCX) [file pone.0239393.s002.docx]

**S2 Table:** **HBV Transplants and Co-infection with HCV or HDV by year, frequency and percent.**

| **Year** | **2010** | **2011** | **2012** | **2013** | **2014** | **2015** | **2016** | **2017** | **2018** | **2019** |
| --- | --- | --- | --- | --- | --- | --- | --- | --- | --- | --- |
| **HBV*** | 232  (71.4%) | 254  (71.8%) | 210  (71%) | 242  (74.9%) | 230  (71%) | 217  (72.8%) | 235  (74.6%) | 255  (78%) | 294  (85%) | 282  (79.5%) |
| **HBV***  **+HCV** | 88  (27.1%) | 95  (26.8%) | 80  (27.0%) | 71  (22%) | 82  (25.3%) | 75  (25.2%) | 65  (20.6%) | 68  (20.8%) | 42  (12.1%) | 58  (16.3%) |
| **HBV***  **+HDV** | 5  (1.5%) | 5  (1.4%) | 6  (2%) | 10  (3.1%) | 12  (3.7%) | 6  (2%) | 15  (4.8%) | 4  (1.2%) | 10  (2.9%) | 15  (4.2%) |
| **Total** | 325 | 354 | 296 | 323 | 324 | 298 | 315 | 327 | 346 | 355 |

***+ALD**
