# Supplementary material for: Causes and trends in liver disease and hepatocellular carcinoma among men and women who received liver transplants in the U.S., 2010-2019
Source: PLoS One. 2020 Sep 18;15(9):e0239393. doi: 10.1371/journal.pone.0239393 (PMC7500679; doi:10.1371/journal.pone.0239393)
Supplement: S3 Table — (DOCX) [file pone.0239393.s003.docx]

**S3 Table . Underlying etiology of HCC among adult liver transplant recipients from 2010 to 2019, by frequency**

| **Frequency** | **2010** | **2011** | **2012** | **2013** | **2014** | **2015** | **2016** | **2017** | **2018** | **2019** |
| --- | --- | --- | --- | --- | --- | --- | --- | --- | --- | --- |
| **HCV** | 1108 | 1180 | 1283 | 1257 | 1339 | 1407 | 1204 | 1227 | 1101 | 873 |
| **ALD** | 115 | 147 | 159 | 146 | 163 | 191 | 226 | 276 | 290 | 315 |
| **NAFLD** | 82 | 114 | 129 | 149 | 174 | 229 | 287 | 306 | 380 | 374 |
| **Cholestatic** | 31 | 30 | 28 | 33 | 20 | 38 | 23 | 39 | 28 | 38 |
| **HBV** | 139 | 182 | 149 | 154 | 152 | 154 | 154 | 128 | 162 | 173 |
| **Cryptogenic** | 49 | 52 | 48 | 51 | 39 | 42 | 52 | 56 | 59 | 40 |
| **Autoimmune** | 14 | 19 | 15 | 20 | 21 | 23 | 22 | 21 | 16 | 21 |
| **Metabolic** | 17 | 17 | 10 | 24 | 17 | 19 | 25 | 21 | 21 | 21 |
| **Budd-Chiari** | 1 | 1 | 1 | 2 | 1 | 1 | 6 | 2 | 2 | 5 |
| **Miscellaneous** | 1 | 0 | 0 | 1 | 3 | 1 | 3 | 1 | 2 | 0 |
| **Other/Unspecified Causes of HCC** | 77 | 62 | 68 | 91 | 100 | 80 | 63 | 99 | 108 | 132 |
| **Total** | 1634 | 1804 | 1890 | 1928 | 2029 | 2185 | 2065 | 2176 | 2169 | 1992 |
