# Supplementary material for: Causes and trends in liver disease and hepatocellular carcinoma among men and women who received liver transplants in the U.S., 2010-2019
Source: PLoS One. 2020 Sep 18;15(9):e0239393. doi: 10.1371/journal.pone.0239393 (PMC7500679; doi:10.1371/journal.pone.0239393)
Supplement: S4 Table — (DOCX) [file pone.0239393.s004.docx]

**S4 Table: Etiology of liver disease among liver transplant recipients with or without HCC by year, frequency and percentage.**

| **Number**  **Percentage** | **2010** | **2011** | **2012** | **2013** | **2014** | **2015** | **2016** | **2017** | **2018** | **2019** |
| --- | --- | --- | --- | --- | --- | --- | --- | --- | --- | --- |
| **HCV*** | 1440  25.13 | 1338  23.05 | 1330  23.21 | 1271  21.47 | 1284  20.71 | 1061  16.21 | 1043  14.35 | 897  11.99 | 756  9.83 | 690  8.27 |
| **HCV* + HCC** | 1108  19.33 | 1180  20.32 | 1283  22.39 | 1257  21.23 | 1339  21.60 | 1407  21.49 | 1204  16.57 | 1227  16.40 | 1101  14.32 | 873  10.46 |
| **ALD** | 612  10.68 | 617  10.63 | 595  10.38 | 665  11.23 | 754  12.16 | 967  14.77 | 1270  17.47 | 1418  18.95 | 1630  21.20 | 2084  24.97 |
| **ALD + HCC** | 115  2.01 | 147  2.53 | 159  2.77 | 146  2.47 | 163  2.63 | 191  2.92 | 226  3.11 | 276  3.69 | 290  3.77 | 315  3.77 |
| **NAFLD** | 437  7.63 | 420  7.23 | 466  8.13 | 549  9.27 | 618  9.97 | 683  10.43 | 977  13.44 | 1064  14.22 | 1164  15.14 | 1421  17.03 |
| **NAFLD + HCC** | 82  1.43 | 114  1.96 | 129  2.25 | 149  2.52 | 174  2.81 | 229  3.50 | 287  3.95 | 306  4.09 | 380  4.94 | 374  4.48 |
| **Cholestatic** | 450  7.85 | 514  8.85 | 436  7.61 | 459  7.75 | 460  7.42 | 513  7.84 | 618  8.50 | 612  8.18 | 563  7.32 | 603  7.23 |
| **Cholestatic + HCC** | 31  0.54 | 30  0.52 | 28  0.49 | 33  0.56 | 20  0.32 | 38  0.58 | 23  0.32 | 39  0.52 | 28  0.36 | 38  0.46 |
| **HBV**** | 186  3.25 | 172  2.96 | 147  2.56 | 169  2.85 | 172  2.77 | 144  2.20 | 161  2.22 | 199  2.66 | 184  2.39 | 182  2.18 |
| **HBV** + HCC** | 139  2.43 | 182  3.13 | 149  2.60 | 154  2.60 | 152  2.45 | 154  2.35 | 154  2.12 | 128  1.71 | 162  2.11 | 173  2.07 |
| **Cryptogenic** | 221  3.86 | 218  3.75 | 160  2.79 | 157  2.65 | 141  2.27 | 168  2.57 | 212  2.92 | 189  2.53 | 211  2.74 | 219  2.62 |
| **Cryptogenic + HCC** | 49  0.85 | 52  0.90 | 48  0.84 | 51  0.86 | 39  0.63 | 42  0.64 | 52  0.72 | 56  0.75 | 59  0.77 | 40  0.48 |
| **Autoimmune** | 134  2.34 | 136  2.34 | 156  2.72 | 147  2.48 | 126  2.03 | 154  2.35 | 190  2.61 | 182  2.43 | 199  2.59 | 213  2.55 |
| **Autoimmune + HCC** | 14  0.24 | 19  0.33 | 15  0.26 | 20  0.34 | 21  0.34 | 23  0.35 | 22  0.30 | 21  0.28 | 16  0.21 | 21  0.25 |
| **Metabolic** | 131  2.29 | 153  2.64 | 157  2.74 | 140  2.36 | 164  2.65 | 152  2.32 | 193  2.66 | 203  2.71 | 179  2.33 | 222  2.66 |
| **Metabolic + HCC** | 17  0.30 | 17  0.29 | 10  0.17 | 24  0.41 | 17  0.27 | 19  0.29 | 25  0.34 | 21  0.28 | 21  0.27 | 21  0.25 |
| **Acute hepatic necrosis (non-HBV/HCV)** | 180  3.14 | 149  2.57 | 137  2.39 | 146  2.47 | 155  2.50 | 143  2.18 | 153  2.11 | 150  2.00 | 165  2.15 | 179  2.14 |
| **Other/Unspecified Causes of HCC** | 77  1.34 | 62  1.07 | 68  1.19 | 91  1.54 | 100  1.61 | 80  1.22 | 63  0.87 | 99  1.32 | 108  1.40 | 132  1.58 |
| **Graft Failure** | 10  0.17 | 14  0.24 | 41  0.72 | 65  1.10 | 88  1.42 | 100  1.53 | 125  1.72 | 104  1.39 | 148  1.93 | 142  1.70 |
| **Benign Hepatic Tumors** | 37  0.65 | 31  0.53 | 28  0.49 | 29  0.49 | 33  0.53 | 57  0.87 | 50  0.69 | 67  0.90 | 57  0.74 | 103  1.23 |
| **Other Malignant Liver Tumors** | 46  0.80 | 52  0.90 | 46  0.80 | 58  0.98 | 59  0.95 | 73  1.12 | 45  0.62 | 69  0.92 | 73  0.95 | 93  1.11 |
| **Budd Chiari + HCC** | 1  0.02 | 1  0.02 | 1  0.02 | 2  0.03 | 1  0.02 | 1  0.02 | 6  0.08 | 2  0.03 | 2  0.03 | 5  0.06 |
| **Budd-Chiari** | 26  0.45 | 26  0.45 | 13  0.23 | 20  0.34 | 12  0.19 | 21  0.32 | 16  0.22 | 24  0.32 | 24  0.31 | 25  0.30 |
| **Miscellaneous** | 15  0.26 | 21  0.36 | 25  0.44 | 28  0.47 | 23  0.37 | 29  0.44 | 35  0.48 | 24  0.32 | 31  0.40 | 32  0.38 |
| **Miscellaneous + HCC** | 1  0.02 | 0  0.00 | 0  0.00 | 1  0.02 | 3  0.05 | 1  0.02 | 3  0.04 | 1  0.01 | 2  0.03 | 0  0.00 |
| **Other/Unspecified** | 172  3.00 | 141  2.43 | 104  1.81 | 90  1.52 | 82  1.32 | 97  1.48 | 115  1.58 | 105  1.40 | 134  1.74 | 145  1.74 |
| **Total** | 5731 | 5806 | 5731 | 5921 | 6200 | 6547 | 7268 | 7483 | 7687 | 8345 |

***+ALD**

****+HCV/HDV/ALD**
